# Compassionate Behavior of Clinical Faculty: Associations with Role Modelling and Gender Specific Differences

**DOI:** 10.5334/pme.1481

**Published:** 2025-03-24

**Authors:** Rosa Bogerd, Milou E. W. M. Silkens, Benjamin Boerebach, José P. S. Henriques, Kiki M. J. M. H. Lombarts

**Affiliations:** 1 Professional Performance and Compassionate Care Research Group, Department of Medical Psychology, Amsterdam UMC, the Netherlands; 2 University of Amsterdam, Amsterdam, the Netherlands; 3 Quality of Care program, Amsterdam Public Health Research Institute, Amsterdam, the Netherlands; 4 Erasmus School of Healthcare Policy & Management, Erasmus University, Rotterdam, the Netherlands; 5 St. Antonius Hospital Emergency Medicine Department, Nieuwegein, The Netherlands; 6 Department of Cardiology, Amsterdam UMC, Amsterdam, the Netherlands; 7 Professional Performance and Compassionate Care Research Group, Department of Medical Psychology, Amsterdam UMC, University of Amsterdam, Amsterdam, the Netherlands

## Abstract

**Introduction:**

For future doctors, learning compassion skills is heavily dependent on female and male faculty’s role modelling in practice. As such, more insight into the relationships between faculty’s compassionate behavior, faculty gender and role modelling is needed.

**Methods:**

In this cross-sectional survey, we analyzed 12416 resident evaluations of 2399 faculty members across 22 Dutch hospitals. The predictor variables were: observed compassionate behavior, faculty gender (reference category: female), and an interaction term between those two. Our outcome variables were: person, teacher and physician role model. All variables, except for faculty gender, were scored on a 7-point Likert scale ranging from 1 “totally disagree” to 7 “totally agree”.

**Results:**

Female faculty scored slightly but significantly higher (M = 6.2, SD = 0.7) than male faculty (M = 5.9, SD = 0.6) on observed compassionate behavior. Observed compassionate behavior was significantly positively associated with being seen as a role model teacher (b = 0.695; 95% CI = 0.623 – 0.767), physician (b = 0.657; 95% CI = 0.598 – 0.716) and person (b = 0.714; 95% CI = 0.653 – 0.775). Male gender showed significant negative associations with role model teacher (b = –0.847; 95% CI = –1.431 – –0.262), physician (b = –0.630, 95% CI = –1.111 – –0.149) and person (b = –0.601, 95% CI = –1.099 – –0.103). The interaction term showed positive significant associations with role model teacher (b = 0.157, 95% CI = 0.061 – 0.767), physician (b = 0.116, 95% CI = 0.037 – 0.194) and person (b = 0.102, 95% CI = 0.021 – 0.183).

**Discussion:**

Dutch residents, in general, observed their faculty to be compassionate towards patients and families and faculty’s observed compassionate behavior is related to being seen as a role model. However, male faculty benefit more from demonstrating compassion, as it has a greater positive influence on their perceived role model status compared to female faculty.

## Introduction

In current times where healthcare systems are facing huge patient demands and workforce shortages at the same time, patients frequently experience a lack of compassionate behavior from their healthcare professionals [1,2,3,4]. This is worrisome, as compassion is a crucial element of high quality patient care. Patients who experience their care as compassionate are less anxious, have lower stress and pain levels, and even report quicker wound healing and other favorable clinical outcomes [5,6,7]. Perhaps even more disturbing, the absence of compassion in patient care has been associated with an increased risk of medical errors and physician burnout [7,8,9] and higher healthcare spending in the long run [7,10].

Faculty may express compassionate behavior through both small and grand gestures in medical practice. Small gestures include, for example, putting a hand on a patient’s shoulder or verbally expressing one’s understanding of the patient’s feelings, whereas visiting the patient more often than usual is an example of a grand gesture [11,12]. Caring for patients with compassion is not self-evident. Some faculty show more compassion than others [13,14,15]. Research suggests that there are individual attributes, experiences and characteristics that can be associated with the variation in clinicians’ expressions of compassionate behavior towards patients [15,16,17]. Gender may be such a characteristic. Although female faculty are often expected to be more compassionate, conforming to socially and historically defined gender roles [18,19], research on the difference in compassionate behavior between male and female faculty is not unanimous. A 2022 systematic review by Pavlova and colleagues [17] found a comparable number of studies favoring female faculty and studies showing no effect for gender on compassionate behavior (respectively 20 versus 18 studies); only three studies found male faculty to be more compassionate [17]. Two-thirds of the studies included in the review used quantitative methods, but of those, almost all were self-report studies. There is little to no quantitative research on faculty’s compassionate behavior as observed by residents. As the transfer of compassion skills to future doctors is heavily dependent on faculty’s (positive) role modelling in practice [16,20,21,22], understanding whether and to what extent residents actually observe female and male faculty’s compassionate behavior in practice is essential.

Role modelling is the overarching activity that includes everything faculty do in their being and acting as professionals [23,24]. For residents a role model is a supervisor whose skills and behaviors they desire to emulate [25] which, in addition to formal curricula, makes role modeling a powerful strategy to instill essential professional competencies in young doctors [26]. Medical education research commonly distinguishes three role model typologies: teacher, physician and person role models [23,24]. Teacher role model qualities include, for example, a student-centered approach and effective communication and feedback. A physician role model is admired for their medical knowledge and skills, sound clinical reasoning and clear communication with patients and staff. Person role models know how to maintain effective interpersonal relationships, are enthusiastic and promote healing through qualities such as compassion, honesty and integrity [24]. Residents mentioned compassion as an important attribute for clinical and person models, but not for the teacher role specifically [24,27]. Additionally, previous work found male role models to be generally described as more admirable and more likely to be admired for their personalities than for their professionalism [28]. Research on the relationship between compassion and role modeling is scarce, outdated and shows contradictory results [13,20,29,30] and the effect of gender on this relationship has not been studied yet. Given that role modelling is crucial for instilling compassion skills [16,20,21,22], that some faculty fail to role model compassion in practice [13,14] and that it is largely a matter of chance which role models residents encounter during their training [31], more insight into the effect of faculty gender on the relationship is needed.

This study aims to answer the following research questions: 1) To what extent do residents observe faculty showing compassionate behavior towards patients, and are these perceptions equal for female and male faculty? 2) To what extent do faculty’s observed compassionate behavior towards patients and faculty gender predict them ‘being seen as a role model’ by residents, and does this differ for female and male faculty?

## Methods

### Study design and setting

In the Netherlands, the eight academic medical centers provide residency training in collaboration with multiple affiliated teaching hospitals. Within these hospitals, residency training is the joint responsibility of the teaching faculty group led by a program director. The duration of residency training varies between specialties (three to six years) [32]. As the quality of medical workplace training ultimately determines the quality of patient care [21], regular evaluation of faculty’s teaching qualities by their residents is incorporated in medical training quality and improvement cycles.

In the Netherlands, the *System for Evaluation of Teaching Qualities* (SETQ) is a widely used and well researched teaching evaluation tool [33,34,35]. With the latest validated 31 item questionnaire, residents evaluate their faculty’s qualities within six teaching domains: learning climate, professional attitude towards residents, learner centeredness, evaluation of residents, feedback to residents and professional practice management. Apart from these teaching domains, the SETQ includes two separate items in which residents score faculty on: “adhering to professional practice standards” and “demonstrating compassion toward patients and their families” [33]. For a full overview of the SETQ questionnaire see supplement I ‘SETQ Questionnaire’.

### Study participants and data collection

To address our study aims, we gathered empirical data between August 2020 and March 2023. Data were collected using the SETQ questionnaire, via the online Perito Professional Performance platform [36]. Residents from programs of various specialties were included. We decided to also include ‘junior doctors not in residency training’^1^ and fellows. The former because, although they are not enrolled in postgraduate training, they have similar tasks to residents and the latter because they are still in training albeit as medical specialists. In this paper, residents refers to all of those included in this study.

In the email invitation for the web-based SETQ questionnaire residents were told to evaluate only those faculty members whom they regularly work with and whom they feel they can evaluate accurately. The email further informed residents about the formative purpose and use of the evaluations, as well as the purpose of the research, and stressed that participation was anonymous and voluntary.

### Measures

Our outcome variables were the three types of role-modelling, measured by the items “During my residency training, this attending supervisor generally 1) is a role model to me as a teacher/ supervisor, 2) is a role model to me as a physician, 3) is a role model to me as a person”. The main predictor in our study was ‘observed compassionate behavior towards patients’. This variable was measured using the item “During my residency training, this attending supervisor generally demonstrates compassion towards patients and their families”. Our second predictor variable was gender (male/ female). Both our main predictor, observed compassionate behavior towards patients, as well as all three role-modelling items were scored on a 7-point Likert scale: 1 = “totally disagree”, 2 = “disagree”, 3 = “somewhat disagree”, 4 = “neutral”, 5 = “somewhat agree”, 6 = “agree”, 7 = “totally agree” or “not applicable” [37].

### Statistical analyses

Since faculty were evaluated by multiple residents, we aggregated and analyzed our data on the level of the faculty member. Before aggregation, we removed 239 duplicate cases referring to residents who evaluated the same faculty member twice or more at one moment in time. We included only faculty whose SETQ scores were based on at least 3 unique resident evaluations to assure the reliability of the included scores [34].^2^ Furthermore, when faculty had participated in multiple evaluation cycles (e.g. in 2021 and in 2023), we included only the scores of their most recent evaluation cycle. Finally, for the variable “faculty gender”the options were male, female, and ‘other’. While suboptimal from a research perspective, evaluations used in medical training quality and improvement cycles often offer limited gender options to protect privacy. In our study, the ‘other’ option was set to missing because the category was relatively small (8.4%) and could encompass multiple meanings (e.g. a gender other than male or female or the wish not to provide their gender).

The first aim of this study was to explore to what extent residents perceive faculty to behave compassionately towards their patients and to shed light on any gender-related differences between faculty. To this end we employed descriptive statistics and conducted an independent samples t-test, using faculty gender as our grouping variable.

Our second goal was to investigate to what extent faculty’s compassionate behavior towards patients predicts them being seen as a role model and whether and how this differs for female and male faculty. For this purpose, we built a multivariate general linear model (GLM). All assumptions for a multivariate GLM were met.^3^ Within our model we used multiple outcome variables (the three types of role models) simultaneously with the same set of predictors (observed compassionate behavior towards patients and faculty gender (reference category = female)). We also entered an interaction term (observed compassionate behavior*faculty gender) in the model, as a third predictor. As the nature of our study is fairly explorative, a significance level of α < 0.05 was used to interpret the various effects found. As a sensitivity analysis, we additionally explored whether there were any differences in the scores given by male versus female residents on all our predictor and outcome variables. We also checked whether the scores of female residents were different for male and female faculty and vice versa. All analyses were performed using SPSS Statistics V.28.

### Ethical approval

The institutional ethical review board of the Amsterdam UMC of the University of Amsterdam provided a waiver declaring the Medical Research Involving Human Subjects Act (WMO) did not apply to the current study (reference number 2023.0844). This study complies with the EU GDPR guidelines.

## Results

In total, 2399 faculty members representing 155 departments in 22 hospitals were scored based on 12416 resident evaluations, of mostly female residents (64.0%) Most faculty were male (51.1%), non-surgical specialists (38.8%) and worked in a university hospital (55.4%) The median number of resident evaluations per supervisor was 4. (See Table 1 for participant characteristics).

**Table 1:** Characteristics of study participants.


DEMOGRAPHIC CHARACTERISTICS	N	FREQUENCY (%)

Number of residents participated	2425	

Gender (residents)		

Male	783	32.3%

Female	1390	57.3%

Missing	252	10.4%

Number of resident evaluations	12416	

Number of faculty evaluated	2399	

Median number of evaluations per faculty	4	

Gender (faculty)		

Male	1124	51.1%

Female	1077	44.9%

Missing	198	8.3%

Type of institute (faculty)		

University hospital	1326	55.3%

General hospital	285	11.9%

Independent treatment center	21	0.9%

Mental healthcare center	21	0.9%

Tertiary referral hospital	671	28.0%

Missings	75	3.1%

Specialty category (faculty)^a^		

Non-surgical	932	38.8%

Surgical	753	31.4%

Supportive	395	16.5%

Non-medical	152	6.3%

Missings	167	7.0%


^a^ For a detailed description of the specialty categories see Supplement III ‘Categorization of specialty programs’.

On all included variables supervisors scored above a mean of 5.6 on a 7-point Likert scale. Female (F) faculty scored slightly but significantly higher than male (M) faculty on observed compassionate behavior towards patients (F = 6.2 (SD = 0.6) versus M = 5.9 (SD = 0.7)) and on all types of role modelling (teacher: F = 5.8 (SD = 0.8) versus M = 5.7 (SD = 0.9), physician: F = 6.0 (SD = 0.7) versus M = 5.9 (SD = 0.8) and person: F = 5.8 (SD = 0.7) versus M = 5.6 (SD = 0.8)). The highest score found is female faculty’s mean score on observed compassionate behavior (6.2, SD = 0.6) and the lowest is male faculty’s mean score on person role model (5.6, SD = 0.8). (See Table 2 for mean and median scores and p values for the independent samples t-test). Based on visual inspection of our data, resident gender did not seem to affect these scores. (See supplement II ‘Sensitivity analyses resident gender’.)

**Table 2:** Mean and median scores of resident ratings of observed compassionate behavior and role-modelling and t-test p value (α < 0.05).


MAIN VARIABLES	ALL FACULTY		FEMALE FACULTY		MALE FACULTY		
		
	MEAN (sd)	MEDIAN (25–75 PERCENTILE)	MEAN (sd)	MEDIAN (25–75 PERCENTILE)	MEAN (sd)	MEDIAN (25–75 PERCENTILE)	INDEPENDENT t-TEST

**Observed compassionate behavior**	6.1 (0.6)	6.2 (5.7–6.5)	6.2 (0.6)	6.3 (6.0–6.7)	5.9 (0.7)	6.0 (5.6–6.4)	t = –9.6, p < 0.001

**Teacher role model**	5.7 (0.9)	5.9 (5.3–6.3)	5.8 (0.8)	6.0 (5.3–6.3)	5.7 (0.9)	5.8 (5.3–6.3)	t = –2.5, p = 0.013

**Physician role model**	5.9 (0.7)	6.0 (5.6–6.4)	6.0 (0.7)	6.0 (5.7–6.5)	5.9 (0.8)	6.0 (5.5–6.3)	t = –3.4, p < 0.001

**Person role model**	5.7 (0.8)	5.8 (5.3–6.3)	5.8 (0.7)	6.0 (5.4–6.3)	5.6 (0.8)	5.8 (5.3–6.3)	t = –5.4, p < 0.001


We found significant positive associations between observed compassionate behavior from faculty towards patients and them being seen as a role model teacher (b = 0.695; 95% CI = 0.623 – 0.767), physician (b = 0.657; 95% CI = 0.598 – 0.716) and person (b = 0.714; 95% CI = 0.653 – 0.775). Our second predictor variable, faculty gender (reference category = female) was significantly associated with all three role model types: teacher (b = –0.847; 95% CI = –1.431 – –0.262), physician (b = –0.630, 95% CI = –1.111 – –0.149) and person (b = –0.601, 95% CI = –1.099 – –0.103). Finally, the interaction term between observed compassionate behavior and faculty gender showed small but significant associations with the teacher (b = 0.157, 95% CI = 0.061 – 0.767), the physician (b = 0.116, 95% CI = 0.037 – 0.194) and the person role model (b = 0.102, 95% CI = 0.021 – 0.183). (Table 3). Visual inspection of descriptive analyses revealed no indication of an effect of resident gender (see supplement II ‘Sensitivity analyses resident gender’). For a more detailed summary of the results of our multivariate GLM see supplement IIII ‘Mutivariate GLM summary’.

**Table 3:** Unadjusted multivariate general linear model (GLM) predicting faculty being seen as a role model by residents according to their gender and their observed compassionate behavior towards patients and families.


	B (SE)	95% CI		*P*

OBSERVED COMPASSIONATE BEHAVIOR		*LL*	*UL*	
	
Observed compassionate behavior on teacher role model	0.695 (0.037)	0.623	0.767	<0.001

Observed compassionate behavior on physician role model	0.657 (0.030)	0.598	0.716	<0.001

Observed compassionate behavior on person role model	0.714 (0.031)	0.653	0.775	<0.001

Faculty gender^a^				

Faculty gender on teacher role model	–0.847 (0.298)	–1.431	–0.262	0.005

Faculty gender on physician role model	–0.630 (0.245)	–1.111	–0.149	0.010

Faculty gender on person role model	–0.601(0.254)	–1.099	–0.103	0.018

Observed compassionate behavior*Faculty gender				

Interaction on teacher role model	0.157 (0.049)	0.061	0.767	0.001

Interaction on physician role model	0.116 (0.040)	0.037	0.194	0.004

Interaction on person role model	0.102 (0.041)	0.021	0.183	0.014


^a^ The reference category for this variable was female.

## Discussion

### Main findings

This study quantitively explored how residents perceive their faculty’s compassionate behaviors towards patients, and whether these observed behaviors were associated with being better role models. This is especially important as role modelling is an important strategy to transfer compassion related skills to residents. We found that Dutch residents, in general, observed their faculty to be compassionate towards patients (mean = 6.1 on a 7-point scale; SD = 0.6), and that female (F) faculty performed slightly but significantly better than their male (M) peers (F = 6.2 (SD = 0.7) versus M = 5.9 (SD = 0.6)). Although these differences may not be immediately apparent in practice, the results of this study are consistent in showing female faculty outperforming their male peers on all variables included, with the largest difference between males and females found on observed compassionate behavior. Additionally, residents perceived the more compassionate supervisors as better role model teachers, role model physicians and role model persons. The interaction effect between observed compassionate behavior and faculty gender indicated that when male faculty showed compassion, this reflected on them being seen as a role model more positively than when female faculty demonstrated compassion. As far as this study was able to detect, resident gender did not affect these results.

### Explanation of findings

The mean score of 6.1 for faculty’s observed compassionate behaviors as measured in this study suggests that residents in general perceive faculty to show compassion towards patients and families. This score is comparable to previously reported findings on compassionate behaviors in the context of evaluating faculty’s clinical – instead of teaching – performance [38]. When asked about faculty’s patient-centeredness competencies, which included the item “Shows compassion to patients”, residents rated their faculty with an average of 4.43 on a 5-point Likert scale [38]. Peer faculty and other healthcare professionals rated faculty’s compassionate behavior slightly higher than residents did and provided scores between 4.45 and 4.55 on a 5-point Likert scale [38].

The average role modelling scores found in our study ranged from 5.7 (person and teacher) to 5.9 (physician). These scores are comparable to the findings in a 2012 SETQ study, which showed similar results on all three types of role modelling – the physician role model type also slightly outscoring the others [23]. The most recent SETQ study, which was performed among US surgeons, also reported scores comparable to our findings, although the highest scores were found both on the physician and the teacher role model type [39]. Notably, the many SETQ studies performed amongst different specialties, in various healthcare settings and contexts around the world, show that overall, teaching faculty score well above a 3.5 on a 5-point Likert scale, and well above a 5.0 when a 7-point scale is used on all items and teaching domains [33,37,39,40,41].

The found relationship between compassionate behavior and role modelling in this study underscores previous qualitative findings that compassionate behavior, according to residents, is an important attribute of being seen as a role model [24,27]. As residents also observe their faculty’s expressions of compassionate care in practice, together these findings endorse the notion that role modelling might indeed be a powerful strategy to instill compassionate care skills in residents [42]. Previous research, however, showed that residents’ perceptions and ideas about what compassionate care entails may differ from patients’ experiences with and expectations of compassionate care. Based on interviews with residents and patients, Debets et al. (2024) reported that *“residents often perceived that assessing patients’ compassion needs comes down to the adage ‘do unto others as you would have them do unto you’”*, whereas patients also stressed the importance of being asked about their specific needs [12]. From this viewpoint, it is possible that the main, if not only, strategy to convey compassionate care skills to the next generation of physicians, overlooks crucial elements of compassionate care. Consequently, explicitly educating practicing faculty, residents and medical students about the fundamentals of compassion becomes a necessary additional step. This may be something to address in medical curriculum reform processes as there is strong evidence about the beneficial effects of compassion for both patients and physicians [3,5,7,16,43,44,45].

Based on this quantitative study, it is unclear what exact behavioral differences in compassionate behavior were observed by residents. Other studies have highlighted gender-specific practices suggesting that female physicians display more patient-centered empathetic communication and better psychosocial counselling [18,46,47,48]. These practices resonate with historically female-coded qualities, such as nurturing, expressiveness and relationship building, all of which make up compassionate behaviors [18,19,49]. Notably, in a previous SETQ study we also found higher ratings for female physicians in the more communicative teaching domains of giving residents feedback and displaying professional attitudes to residents [50]. Some might argue that given historical assumptions in the medical field and Western society about female physicians being expected to show compassion towards patients, more so than male peers, the variations we discovered in our study might actually be more pronounced in reality. Thus, though residents might already anticipate compassion from their female supervisors, our findings still show a notable difference favoring women.

Lastly, in the interaction between observed compassionate behavior and gender we found that showing compassion reflects more positively on male supervisors than on female supervisors, i.e. males expressing compassion are rewarded by being seen as role models more than their female peers displaying compassion. Although the found effect was small and therefore perhaps of limited relevance in practice, this finding aligns with the general idea that male physicians are more admired for showing humanistic behaviors because showing these behaviors goes against previously mentioned gendered expectations [18,28]. In previous studies, using patient evaluations, patient-centered behavior in male physicians was associated with higher levels of greater overall performance than in female physicians [47,51].

### Implications for clinical education, research and policy

Our study findings show that residents do observe faculty’s compassionate behavior towards patients in practice and that this observed behavior is indeed associated with being a better role model. The found association suggests that using role modelling as a teaching strategy for compassion, and maybe broader humanistic skills, might indeed be effective. As the effect of observed compassionate behavior is stronger for male physicians, and they are thus ‘rewarded’ more for being compassionate towards patients than their female peers, this study contributes to the growing understanding that the medical profession is not gender-neutral [18,19,52,53]. On the whole, this study shows that compassion is being seen and appreciated by residents and could therefore increase knowledge among faculty about the impact of their behaviors towards patients and families.

Which role models residents encounter during their medical training is largely a matter of chance: faculty may vary in how they express compassion towards patients and families [54,55] and experiences with negative role models lacking humanistic behaviors have been reported [13,14,31,56]. Variation in staff groups, e.g. with regards to gender, may therefore be a practical and potentially beneficial strategy for transferring a range of compassion skills to future doctors. In addition, future research could be aimed at revealing and understanding differences in expressing compassionate behavior between (female and male) faculty in a qualitative way, for example by analyzing the narrative feedback in teaching qualities assessments [57] or by using observational methods. These insights can be used to inform teacher training courses as well as courses on compassion in the (post-graduate) medical curriculum. Lastly, in line with previous empathy research [58], it may be interesting to explore whether there are differences in (observed) compassionate behavior across faculty members from various medical specialties.

### Strengths and limitations

This study’s multicenter approach, which included both academic and non-academic teaching hospitals, ensures our sample’s validity of Dutch clinical teaching faculty. The minimum number of 3 and median number of 4 resident evaluations per faculty guarantee the reliability of teaching faculty scores [59]. Also, we included approximately one third of all Dutch medical residents with a gender division resembling the one in the population [60]. This strengthens us in our conviction that the scores provided are representative of how Dutch residents perceive their supervisors. When interpreting the findings of our study, one should keep in mind the cross sectional nature of this study. Based on previous research [20], we hypothesized that showing compassion towards patients was associated with being seen as a role model in the eyes of residents. The associations found in this study, however, may as well reflect a reverse relationship between these variables. Furthermore, the data of this study were partly gathered during the COVID pandemic, which may have slightly colored our findings. Lastly, although we are familiar with the possibility of a halo effect in survey studies into people’s characteristics [61], previous research reassures us that residents do differentiate between faculty’s (positive) qualities using the SETQ questionnaire [37,62].

## Conclusions

This study explored the relationship between faculty’s observed compassionate behavior towards patients and them being seen as role models by residents. The findings of this study indicate that Dutch residents, in general, observed their faculty to be compassionate towards patients and families and that expressing compassion is indeed related to being seen as a role model. The positive effect of observed compassionate behavior on being seen as a role model was stronger for male faculty than for female faculty. This suggests that although showing compassion towards patients reflects positively on all faculty in terms of role modelling, residents admire male faculty more for showing compassionate behaviors than female faculty. As far as this study was able to detect, resident gender did not affect our findings. The results of our study may bring more awareness amongst teaching faculty of the importance of showing compassion to patients in training residents to become compassionate care givers. Although role modelling compassionate behaviors can be an effective teaching strategy, the importance and power of compassion should also be made part of the formal medical curriculum.

## Additional Files

The additional files for this article can be found as follows:

10.5334/pme.1481.s1 Suppplement I. Evaluation of Teaching Qualities (Setq) Questionnaire.

10.5334/pme.1481.s2 Suppplement II. Sensitivity Analyses Resident Gender.

10.5334/pme.1481.s3 Suppplement III. Categorization of Specialty Programs.

10.5334/pme.1481.s4 Suppplement IIII. Multivariate Glm Summary.

## Footnotes

In the Netherlands, medicine graduates often begin their careers as “junior doctors not in training.” In this role, they perform many of the same tasks as residents but are not enrolled in a formal specialty training program. This period allows them to gain valuable clinical experience and build a professional network, which can help them to secure a spot in residency training later on.

In a previous confirmatory factor analysis study of the SETQ, generalizability analysis showed that only two or three evaluations per supervisor was enough for high reliability levels: *“The generalizability analysis, that was conducted to reveal if the trainee evaluations, aggregated on supervisor level, had high α levels even if just a few trainees completed an evaluation for a specific supervisor, revealed that if only two or three trainees completed an evaluation, α > .89 for all subscales.”*

Although the assumption of the outcome variables being continuous was not strictly met in our model, it is quite common in the field of medical education to use a Multivariate GLM with Likert scale variables as outcome measures. In this context, these variables are typically interpreted as continuous scores.

## References

[1] Dignity Health Survey Finds Majority of Americans Rate Kindness as Top Factor in Quality Health Care. Patients willing to pay more and travel further for kinder treatment; Philadelphia receives top grade as kindest city for health care. (13 November 2013). https://www.dignityhealth.org/about-us/press-center/press-releases/majority-of-americans-rate-kindness (assessed 24 February 2025)

[2] Levinson W, Chaumeton N. Communication between surgeons and patients in routine office visits. Surgery. 1999; 125(2): 127–34. DOI: 10.1016/S0039-6060(99)70255-2 10026744

[3] Malenfant S, Jaggi P, Hayden KA, Sinclair S. Compassion in healthcare: an updated scoping review of the literature. BMC Palliat. Care. 2022; 21(1): 80. DOI: 10.1186/s12904-022-00942-3 35585622 PMC9116004

[4] Zulman DM, Haverfield MC, Shaw JG, Brown-Johnson CG, Schwartz R, Tierney AA, et al. Practices to Foster Physician Presence and Connection With Patients in the Clinical Encounter. JAMA. 2020; 323(1): 70–81. DOI: 10.1001/jama.2019.19003 31910284

[5] Pereira L, Figueiredo-Braga M, Carvalho IP. Preoperative anxiety in ambulatory surgery: The impact of an empathic patient-centered approach on psychological and clinical outcomes. Patient Educ Couns. 2016; 99(5): 733–8. DOI: 10.1016/j.pec.2015.11.016 26654958

[6] Hojat M, Louis DZ, Markham FW, Wender R, Rabinowitz C, Gonnella JS. Physicians’ empathy and clinical outcomes for diabetic patients. Acad. Med. 2011; 86(3): 359–64. DOI: 10.1097/ACM.0b013e3182086fe1 21248604

[7] Trzeciak S, Roberts BW, Mazzarelli AJ. Compassionomics: Hypothesis and experimental approach. Med. Hypotheses. 2017; 107: 92–7. DOI: 10.1016/j.mehy.2017.08.015 28915973

[8] Trzeciak S, Mazzarelli A, Booker C. Compassionomics: The revolutionary scientific evidence that caring makes a difference. Pensacola, FL: Studer Group; 2019.

[9] West CP, Huschka MM, Novotny PJ, Sloan JA, Kolars JC, Habermann TM, Shanafelt TD. Association of perceived medical errors with resident distress and empathy: a prospective longitudinal study. JAMA. 2006; 296(9): 1071–8. DOI: 10.1001/jama.296.9.1071 16954486

[10] Bertakis KD, Azari R. Patient-centered care is associated with decreased health care utilization. JABFM. 2011; 24(3): 229–39. DOI: 10.3122/jabfm.2011.03.100170 21551394

[11] Chochinov HM. Dignity and the essence of medicine: the A, B, C, and D of dignity conserving care. BMJ. 2007; 335(7612): 184–7. DOI: 10.1136/bmj.39244.650926.47 17656543 PMC1934489

[12] Debets MPM, Jansen I, Diepeveen M, Bogerd R, Molewijk BAC, Widdershoven GAM, Lombarts MJMH. Compassionate care through the eyes of patients and physicians: an interview study. PLoS One. 2024; 19(7): e0305007. DOI: 10.1371/journal.pone.0305007 38985731 PMC11236150

[13] Beaudoin C, Maheux B, Cote L, Des Marchais JE, Jean P, Berkson L. Clinical teachers as humanistic caregivers and educators: perceptions of senior clerks and second-year residents. Cmaj. 1998; 159(7): 765–9. 9805021 PMC1232732

[14] Benbassat J, Baumal R. What is empathy, and how can it be promoted during clinical clerkships? Acad. Med. 2004; 79(9): 832–9. DOI: 10.1097/00001888-200409000-00004 15326005

[15] Carmel S, Glick SM. Compassionate-empathic physicians: personality traits and social-organizational factors that enhance or inhibit this behavior pattern. Soc Sci Med. 1996; 43(8): 1253–61. DOI: 10.1016/0277-9536(95)00445-9 8903130

[16] Sinclair S, Norris JM, McConnell SJ, Chochinov HM, Hack TF, Hagen NA, et al. Compassion: a scoping review of the healthcare literature. BMC Palliat Care. 2016; 15: 6–22. DOI: 10.1186/s12904-016-0080-0 26786417 PMC4717626

[17] Pavlova A, Wang CX, Boggiss AL, O’Callaghan A, Consedine NS. Predictors of physician compassion, empathy, and related constructs: a systematic review. J Gen Intern Med. 2022; 1–12. DOI: 10.1007/s11606-021-07055-2 PMC8452146 34545471

[18] Lombarts KM, Verghese A. Medicine is not gender-neutral—She is male. N Engl J Med. 2022; 386: 1284–7. DOI: 10.1056/NEJMms2116556 35353969

[19] Sheffield V, Hartley S, Stansfield RB, Mack M, Blackburn S, Vaughn VM, et al. Gendered Expectations: the Impact of Gender, Evaluation Language, and Clinical Setting on Resident Trainee Assessment of Faculty Performance. J Gen Intern Med. 2022; 37(4): 714–22. DOI: 10.1007/s11606-021-07093-w PMC8904706 34405349

[20] Wear D, Zarconi J. Can compassion be taught? Let’s ask our students. J Gen Intern Med. 2008; 23: 948–53. DOI: 10.1007/s11606-007-0501-0 18612722 PMC2517940

[21] Côté L, Laughrea P-A. Preceptors’ Understanding and Use of Role Modeling to Develop the CanMEDS Competencies in Residents. Acad. Med. 2014; 89(6): 934–9. DOI: 10.1097/ACM.0000000000000246 24871246

[22] Wilcox MV, Orlando MS, Rand CS, Record J, Christmas C, Ziegelstein RC, Hanyok LA. Medical students’ perceptions of the patient-centredness of the learning environment. Perspect. Med. Educ. 2017; 6: 44–50. DOI: 10.1007/S40037-016-0317-X 27987074 PMC5285277

[23] Boerebach BCM, Lombarts KMJMH, Keijzer C, Heineman MJ, Arah OA. The Teacher, the Physician and the Person: How Faculty’s Teaching Performance Influences Their Role Modelling. PLoS One. 2012; 7(3): e32089. DOI: 10.1371/journal.pone.0032089 22427818 PMC3299651

[24] Cruess SR, Cruess RL, Steinert Y. Role modelling—making the most of a powerful teaching strategy. Bmj. 2008; 336(7646): 718–21. DOI: 10.1136/bmj.39503.757847.BE 18369229 PMC2276302

[25] Lombarts KM, Heineman MJ, Arah OA. Good clinical teachers likely to be specialist role models: results from a multicenter cross-sectional survey. PLoS One. 2010; 5(12): e15202. DOI: 10.1371/journal.pone.0015202 21206906 PMC3012058

[26] Jochemsen-van der Leeuw HR, van Dijk N, van Etten-Jamaludin FS, Wieringa-de Waard M. The attributes of the clinical trainer as a role model: a systematic review. Acad. Med. 2013; 88(1): 26–34. DOI: 10.1097/ACM.0b013e318276d070 23165277

[27] Passi V, Johnson S, Peile E, Wright S, Hafferty F, Johnson N. Doctor role modelling in medical education: BEME Guide No. 27. Med. Teach. 2013; 35(9): e1422–e36. DOI: 10.3109/0142159X.2013.806982 23826717

[28] Lindberg O. Gender and role models in the education of medical doctors: a qualitative exploration of gendered ways of thinking. Int J Med Educ. 2020; 11: 31–6. DOI: 10.5116/ijme.5e08.b95b 32007950 PMC7246110

[29] Shapiro J. How do physicians teach empathy in the primary care setting? Acad. Med. 2002; 77(4): 323–8. DOI: 10.1097/00001888-200204000-00012 11953298

[30] Maheux B, Beaudoin C, Berkson L, Côté L, Des Marchais J, Jean P. Medical faculty as humanistic physicians and teachers: the perceptions of students at innovative and traditional medical schools. Med. Educ. 2000; 34(8): 630–4. DOI: 10.1046/j.1365-2923.2000.00543.x 10964210

[31] Reuler JB, Nardone DA. Role modeling in medical education. West J Med. 1994; 160(4): 335–7. 8023482 PMC1022423

[32] Association KRDM. Kaderbesluit Centraal College Medische Specialismen; 2009.

[33] Lombarts KM, Ferguson A, Hollmann MW, Malling B, Arah OA. Redesign of the system for evaluation of teaching qualities in anesthesiology residency training (SETQ Smart). Anesthesiology. 2016; 125(5): 1056–65. DOI: 10.1097/ALN.0000000000001341 27606931

[34] Boerebach BCM, Lombarts KMJMH, Arah OA. Confirmatory Factor Analysis of the System for Evaluation of Teaching Qualities (SETQ) in Graduate Medical Training. Eval Health Prof. 2014; 39(1): 21–32. DOI: 10.1177/0163278714552520 25280728

[35] van der Leeuw R, Lombarts K, Heineman MJ, Arah O. Systematic evaluation of the teaching qualities of Obstetrics and Gynecology faculty: reliability and validity of the SETQ tools. PLoS One. 2011; 6(5): e19142. DOI: 10.1371/journal.pone.0019142 21559275 PMC3086887

[36] Perito Professional Performance [Platform for evaluating physcians’ professional performance]. Available from: https://peritoprofessionalperformance.com/.

[37] Debets MPM, Scheepers RA, Boerebach BCM, Arah OA, Lombarts KMJMH. Variability of residents’ ratings of faculty’s teaching performance measured by five- and seven-point response scales. BMC Med. Educ. 2020; 20(1): 325–334. DOI: 10.1186/s12909-020-02244-9 32962692 PMC7510269

[38] van der Meulen MW, Arah OA, Heeneman S, oude Egbrink MG, van der Vleuten CP, Lombarts KM. When Feedback Backfires: Influences of Negative Discrepancies Between Physicians’ Self and Assessors’ Scores on Their Subsequent Multisource Feedback Ratings. JCEHP. 2021; 41(2): 94–103. DOI: 10.1097/CEH.0000000000000347 34009839

[39] Lewis JM, Yared K, Heidel RE, Kirkpatrick B, Freeman MB, Daley BJ, et al. Emotional intelligence and burnout related to resident-assessed faculty teaching scores. J. Surg. Educ. 2021; 78(6): e100–e11. DOI: 10.1016/j.jsurg.2021.09.023 34750078

[40] Boldaji FT, Amini M, Parvizi MM. Psychometric properties of the Persian version of System for Evaluation of Teaching Qualities by students: A tool for assessing clinical tutors from students’ viewpoint. J. Educ. Health. Promot. 2022; 11: 92–101. DOI: 10.4103/jehp.jehp_1622_20 35573607 PMC9093631

[41] Zhou NJ, Kamil RJ, Hillel AT, Tan M, Walsh J, Russell JO, et al. The role of preoperative briefing and postoperative debriefing in surgical education. Journal of surgical education. 2021; 78(4): 1182–8. DOI: 10.1016/j.jsurg.2020.11.001 33257299

[42] Jochemsen-van der Leeuw HGAR, van Dijk N, van Etten-Jamaludin FS, Wieringa-de Waard M. The Attributes of the Clinical Trainer as a Role Model: A Systematic Review. Acad. Med. 2013; 88(1): 26–34. DOI: 10.1097/ACM.0b013e318276d070 23165277

[43] Goetz JL, Keltner D, Simon-Thomas E. Compassion: an evolutionary analysis and empirical review. Psychol. Bull. 2010; 136(3): 351–74. DOI: 10.1037/a0018807 20438142 PMC2864937

[44] Gaufberg E, Hodges B. Humanism, compassion and the call to caring. Wiley Online Library; 2016. p. 264–6. DOI: 10.1111/medu.12961 26896006

[45] Bendapudi NM, Berry LL, Frey KA, Parish JT, Rayburn WL, editors. Patients’ perspectives on ideal physician behaviors. Mayo Clin Proc. 2006; 81(3): 338–344. DOI: 10.4065/81.3.338 16529138

[46] Roter DL, Hall JA. Physician gender and patient-centered communication: a critical review of empirical research. Annu Rev Public Health. 2004; 25: 497–519. DOI: 10.1146/annurev.publhealth.25.101802.123134 15015932

[47] Hall JA, Gulbrandsen P, Dahl FA. Physician gender, physician patient-centered behavior, and patient satisfaction: A study in three practice settings within a hospital. Patient Educ Couns. 2014; 95(3): 313–8. DOI: 10.1016/j.pec.2014.03.015 24731957

[48] Bertakis KD, Franks P, Azari R. Effects of physician gender on patient satisfaction. J Am Med Womens Assoc (1972). 2003; 58(2): 69–75. 12744418

[49] Eagly AH, Wood W. Social role theory of sex differences. The Wiley Blackwell encyclopedia of gender and sexuality studies. 2016; 1–3. DOI: 10.1002/9781118663219.wbegss183

[50] Arah OA, Heineman MJ, Lombarts KM. Factors influencing residents’ evaluations of clinical faculty member teaching qualities and role model status. Med. Educ. 2012; 46(4): 381–9. DOI: 10.1111/j.1365-2923.2011.04176.x 22429174

[51] Blanch-Hartigan D, Hall JA, Roter DL, Frankel RM. Gender bias in patients’ perceptions of patient-centered behaviors. Patient Educ Couns. 2010; 80(3): 315–20. DOI: 10.1016/j.pec.2010.06.014 20638813

[52] Lukela JR, Ramakrishnan A, Hadeed N, Del Valle J. When perception is reality: Resident perception of faculty gender parity in a university-based internal medicine residency program. Perspect Med Educ. 2019; 8: 346–52. DOI: 10.1007/s40037-019-00532-9 31728840 PMC6904409

[53] Paulus JK, Switkowski KM, Allison GM, Connors M, Buchsbaum RJ, Freund KM, Blazey-Martin D. Where is the leak in the pipeline? Investigating gender differences in academic promotion at an academic medical centre. Perspect Med Educ. 2016; 5: 125–8. DOI: 10.1007/S40037-016-0263-7 27001528 PMC4839010

[54] Cameron RA, Mazer BL, DeLuca JM, Mohile SG, Epstein RM. In search of compassion: a new taxonomy of compassionate physician behaviours. Health Expectations. 2015; 18(5): 1672–85. DOI: 10.1111/hex.12160 24305037 PMC4051859

[55] Baguley SI, Pavlova A, Consedine NS. More than a feeling? What does compassion in healthcare ‘look like’ to patients? Health Expect. 2022; 25(4): 1691–702. DOI: 10.1111/hex.13512 35661516 PMC9327826

[56] Lown BA. A social neuroscience-informed model for teaching and practising compassion in health care. Med Educ. 2016; 50(3): 332–42. DOI: 10.1111/medu.12926 26896018

[57] Klein R, Snyder ED, Koch J, Volerman A, Alba-Nguyen S, Julian KA, et al. Analysis of narrative assessments of internal medicine resident performance: are there differences associated with gender or race and ethnicity? BMC Med. Educ. 2024; 24(1): 1–12. DOI: 10.1186/s12909-023-04970-2 38233807 PMC10795394

[58] Hojat M, Gonnella JS, Nasca TJ, Mangione S, Vergare M, Magee M. Physician empathy: definition, components, measurement, and relationship to gender and specialty. Am. J. Psychiatry. 2002; 159(9): 1563–9. DOI: 10.1176/appi.ajp.159.9.1563 12202278

[59] Lombarts KM, Bucx MJ, Arah OA. Development of a system for the evaluation of the teaching qualities of anesthesiology faculty. Anesthesiology. 2009; 111(4): 709–16. DOI: 10.1097/ALN.0b013e3181b76516 19707115

[60] Capaciteitsorgaan. Capaciteitsplan 2024 tot 2027. Deelrapport 1 Medisch specialismen, klinische technologische specialismen, spoedeisende geneeskunde. Utrecht; 2022.

[61] Nicolau JL, Mellinas JP, Martín-Fuentes E. The halo effect: A longitudinal approach. Ann. Tour. Res. 2020; 83: 102938. DOI: 10.1016/j.annals.2020.102938

[62] Boerebach BCM, Arah OA, Heineman MJ, Lombarts KMJMH. Embracing the Complexity of Valid Assessments of Clinicians’ Performance: A Call for In-Depth Examination of Methodological and Statistical Contexts That Affect the Measurement of Change. Acad. Med. 2016; 91(2): 215–20. DOI: 10.1097/ACM.0000000000000840 26200579

