# Supplementary material for: Compassionate Behavior of Clinical Faculty: Associations with Role Modelling and Gender Specific Differences
Source: Perspect Med Educ. 2025 Mar 24;14(1):118–28. doi: 10.5334/pme.1481 (PMC11951979; doi:10.5334/pme.1481)
Supplement: Suppplement I. — Evaluation of Teaching Qualities (Setq) Questionnaire. [file pme-14-1-1481-s1.pdf]

SUPPPLEMENT I. EVALUATION OF TEACHING QUALITIES (SETQ) QUESTIONNAIRE

You fill in the questions by clicking on one answer per question. For most questions, a 7-point scale is used with the following ratings:

1 = strongly disagree
2 = disagree
3 = somewhat disagree
4 = neutral
5 = somewhat agree
6 = agree
7 = strongly agree

During my specialist training, for this supervisor in general, he/she...

LEARNING CLIMATE (LC)

LC1 ...encourages residents to participate actively in discussions.
LC2 ...stimulate residents to bring up problems.
LC3 ...motivates residents to further study.
LC4 ...stimulate residents to keep up with the literature.
LC5 ...prepares well for teaching presentations and talks.

PROFESSIONAL ATTITUDE TOWARDS ASSISTANTS (PA)
PA1 ...listens attentively to residents.
PA2 ...shows respect towards residents..
PA3 ...is easily approachable during on-calls.
PA4 ...is easily approachable for discussions during routine daytime work.

LEARNER CENTREDNESS (LCn)
LCn1 ...clarifies learning goals for the learning session.
LCn2 ...matches residents’ and supervisors’ learning expectations.
LCn2 ...provides residents with responsibilities based on their abilities.
LCn4...teaches residents how to deal with competing personal/ professional demands.

EVALUATION OF RESIDENTS’ KNOWLEDGE AND SKILLS (E)
E1 ...evaluates residents’ specialty knowledge regularly.
E2 ...evaluates residents’ analytical abilities regularly.
E3 ...evaluates residents’ applications of knowledge in daily practice regularly.
E4 ...evaluates residents’ procedural skills regularly.

FEEDBACK (FB)
FB1 ...gives positive feedback to residents.
FB2 ...gives corrective feedback to residents.
FB3 ...explains why residents are (in)correct.
FB4 ...offers suggestions for improvement.

PROFESSIONAL PRACTICE MANAGEMENT (PP)
PP1 ...teaches residents how to deal with colleagues (residents and supervisors) with questionable or inappropriate practis
PP2 ...teaches organizational aspects of healthcare.
PP3 ...creates awareness of the economic aspects of medical care.

SEPARATE ITEMS
Sm12 ...works adhering to the professional standards.
Sm13a ...demonstrates compassion towards patients and their families.

ROLE MODEL (RM)
Faculty members/supervisors can be role models for residents in a number of ways. Can you indicate whether this faculty member/supervisor is a role model for you in the mentioned roles?

During my training, for this attending/supervisor in general, he/she...

RM17 ...is a role model for me as an educator/supervisor.
RM18 ...is a role model for me as a physician.
RM19 ...is a role model for me as a person.

OVERALL
Overall, I rate this faculty member for his/her teaching qualities as a score ...
Answer on a 10-point scale.

Strengths
Please provide this attending/ supervisor with some feedback comments addressing his/her strengths as a supervisor.

Areas for Improvement
Would you please write down a number of improvement points for this supervisor regarding his/her teaching/supervising qualities?
