# Supplementary material for: Compassionate Behavior of Clinical Faculty: Associations with Role Modelling and Gender Specific Differences
Source: Perspect Med Educ. 2025 Mar 24;14(1):118–28. doi: 10.5334/pme.1481 (PMC11951979; doi:10.5334/pme.1481)
Supplement: Suppplement II. — Sensitivity Analyses Resident Gender. [file pme-14-1-1481-s2.pdf]

SUPPLEMENT II. SENSITIVITY ANALYSES RESIDENT GENDER

Our data provide limited possibilities to correct for resident gender in the context of our current study. In the original non-aggregated dataset we have information on the gender of each resident, albeit that some of this information is missing. As multiple residents evaluate one supervisor, our cleaned dataset is aggregated and this means that each supervisor is evaluated by a mix of male and female residents. The construction of proxy variables for the percentage of male and female evaluations per supervisor is possible, but these kinds of variables are difficult to interpret and their reliability is limited due to the existing missingness in our original data. This is why we chose to conduct descriptive analysis in the original non-aggregated dataset to get an idea of the possible effect of resident gender on our results. The caveat is that we cannot provide any causal information and the data does not allow to draw firm conclusions, so these efforts only allow us to look at the effect of resident gender on our results by eyeballing. In the original dataset, resident gender *only* (slightly) significantly affected the scores that were given on compassionate behavior – female residents scored all supervisors 0.1 higher on this variable than male residents did (please see Table I). Table II presents the scores given to male, female and all supervisors when we split the original dataset in male and female residents. As we were not able to properly control for resident gender in our main analysis, we do not know whether the found differences between male and female supervisors would hold when we would have. Our original data, however, do not provide any clues that resident gender has indeed skewed our findings. Future research is needed to determine the effect of resident gender on the relation between observed compassionate behavior of faculty and them being seen as a role model.

	**Mean score based on all included resident evaluations**	**Mean score given by female residents**	**Mean score given by male residents**
**Compassionate behavior**	M = 6.1 (SD = 0.6)	M = 6.1 (SD = 1.0)	M = 6.0 (SD = 1.0)
**Teacher role model**	M = 5.7 (SD = 0.9)	M = 5.7, (SD = 1.3)	M = 5.7 (SD = 1.3)
**Physician role model**	M = 5.9 (SD = 0.7)	M = 5.9 (SD = 1.1)	M = 5.9 (SD = 1.1)
**Person role model**	M = 5.7 (SD = 0.8)	M = 5.7 (SD = 1.2)	M = 5.7 (SD = 1.2)

Table I. Overview of scores provided by female and male residents in original dataset in comparison with reported mean scores in main manuscript. *Female residents score all supervisors significantly 0.1 higher on compassionate behavior (independent samples t-test: $p<0.001$).*

	Female faculty	Male faculty	Female faculty (scored by female residents)	Male faculty (scored by female residents)	Female faculty (scored by male residents)	Female faculty (scored by male residents)
Compassionate behavior	M = 6.2	M = 5.9	M = 6.2	M = 6.0	M = 6.2	M = 5.9
Teacher role model	M = 5.8	M = 5.7	M = 5.7	M = 5.7	M = 5.8	M = 5.7
Physician role model	M = 6.0	M = 5.9	M = 6.0	M = 5.9	M = 5.9	M = 5.9
Person role model	M = 5.8	M = 5.6	M = 5.8	M = 5.6	M = 5.8	M = 5.7

Table II. Scores given to male and female faculty grouped by all, male and female residents in original dataset (before aggregation). *Although some small variants appear in some of the scores when grouping by resident gender, the overall pattern remains unchanged.*
