# Supplementary material for: Compassionate Behavior of Clinical Faculty: Associations with Role Modelling and Gender Specific Differences
Source: Perspect Med Educ. 2025 Mar 24;14(1):118–28. doi: 10.5334/pme.1481 (PMC11951979; doi:10.5334/pme.1481)
Supplement: Suppplement III. — Categorization of Specialty Programs. [file pme-14-1-1481-s3.pdf]

SUPPLEMENT III. CATEGORIZATION OF SPECIALTY PROGRAMS

**Medical**	**Non-medical**
Internal medicine	Medical Psychology
Cardiology	Pharmacy
Pediatrics	Clinical Physics
Pulmonary diseases	Clinical Chemistry
Gastrointestinal and liver diseases	Mental Health
Neurology	Medical Microbiology and Immunology
Rehabilitation medicine	Sexology
Psychiatry	Special Dentistry
Emergency care	Clinical Neurophysiology
Dermatology	Dentistry
Intensive care	
ICL	**Surgical**
General practitioners	Surgery
Nursing home physicians	Gynecology
Sports medicine	ENT
Clinical geriatrics	Neurosurgery
Rheumatology	Plastic surgery
Allergology	Ophthalmology
	Orthopedics
**Supporting**	Urology
Anesthesiology	Oral surgery
Clinical Genetics	Cardio-thoracic surgery
Pathology	
Radiology	
Radiotherapy	
