# Supplementary material for: Compassionate Behavior of Clinical Faculty: Associations with Role Modelling and Gender Specific Differences
Source: Perspect Med Educ. 2025 Mar 24;14(1):118–28. doi: 10.5334/pme.1481 (PMC11951979; doi:10.5334/pme.1481)
Supplement: Suppplement IIII. — Multivariate Glm Summary. [file pme-14-1-1481-s4.pdf]

SUPPLEMENT IIII. MULTIVARIATE GLM SUMMARY

	df	F	*p*	Partial η^2
**Teacher role model**				
Compassionate behavior	1	1009.93	<0.001	0.315
Faculty gender	1	8.08	0.005	0.004
Compassionate behavior x faculty gender	1	10.36	0.001	0.005
**Physician role model**				
Compassionate behavior	1	1274.51	<0.001	0.368
Faculty gender	1	6.61	0.010	0.003
Compassionate behavior x faculty gender	1	8.35	0.004	0.004
**Person role model**				
Compassionate behavior	1	1361.55	<0.001	0.383
Faculty gender	1	5.60	0.018	0.003
Compassionate behavior x faculty gender	1	6.06	0.014	0.003

Table 4. Multivariate general linear model for teacher, physician and person role model.
